# Gamma-irradiated amniotic membrane graft as an adjuvant to posterior colporrhaphy: an exploratory prospective single-arm clinical trial

**DOI:** 10.1186/s12905-026-04919-9

**Published:** 2026-10-17

**Authors:** Rania Hassan Mostafa Ahmed, Mohab Ahmed Fouad ElRabat, Nourhan Ashraf ElSherbiny, Ihab Fouad Serag Eldin Allam, Ahmed Hesham Abdel Hakim, Ahmed Mohamed Abbas Sadek

**Affiliations:** 1 Department of Obstetrics and Gynecology, Faculty of Medicine, Ain Shams University, Cairo, Egypt; 2 Specialty Registrar Obstetrics and Gynecology, Oxford University Hospital, Oxford city, UK

**Keywords:** Posterior colporrhaphy, Amniotic membrane, Biological graft, Pelvic organ prolapse, POP-Q, Sexual function

## Abstract

**Background:**

Posterior compartment prolapse is a frequent pelvic floor disorder. While conventional posterior colporrhaphy is widely used, the recurrence rates and limitations in terms of tissue support remain concerns. Human amniotic membrane represents a promising biological scaffold with potent regenerative properties. This study aimed to evaluate the feasibility, safety and effectiveness of using amniotic membrane as a graft in posterior colporrhaphy.

**Methods:**

This pilot one-arm clinical trial was conducted on women with posterior compartment prolapse at Ain Shams University Hospital during the period (November 2023- August 2025). Twenty-five patients underwent posterior colporrhaphy using gamma-irradiated amniotic membrane as a graft on the rectovaginal septum. We assessed symptoms score via the Pelvic Organ Prolapse Distress Inventory 6 (POPDI-6), sexual function via the Female Sexual Function Index Questionnaire, and pelvic organ prolapse quantification (POP-Q) preoperatively, and at 3 and 6 months post-operatively, and assessed complication rates.

**Results:**

The AM graft was successfully implanted in all patients. The overall procedure was associated with reduction in POPDI-6 scores from 68.00 ± 14.52 at baseline to 20.67 ± 7.93 at 6 months (*p* < 0.001), with a mean percentage reduction of 68.22% ± 14.54%. The sexual function score improved from 11.75 ± 3.55 to 30.80 ± 2.75. Anatomical assessment revealed that 80% of patients achieved POP-Q stage 0, whereas 20% reached stage I by 6 months. No serious complications (wound dehiscence, visceral injury) were reported; however, vaginitis was common, affecting 76% of patients.

**Conclusion:**

Posterior colporrhaphy with adjuvant gamma-irradiated amniotic membrane graft was associated with some improvement in symptoms, clinical examination, as well as sexual function of the patients. However, this is a one-arm pilot study; further studies are needed to compare this technique with conventional colporrhaphy.

**Trial registration:**

Clinicaltrials.gov NCT06117670, on 31/10/2023.

**Supplementary Information:**

The online version contains supplementary material available at https://doi.org/10.1186/s12905-026-04919-9.

## Background

Pelvic organ prolapse (POP) is the herniation or downward displacement of one or more of the pelvic organs (uterus, vaginal apex, bladder, or bowel) into the vagina; affecting around 40–60% of parous women [1]. POP may affect the anterior compartment, apical compartment, or posterior compartment. Posterior compartment prolapse includes either herniation of the rectum (rectocele) or the small intestine (enterocele). Women with rectocele may experience a bulge in the vagina, and symptoms of obstructed defecation, sometimes requiring splinting or digitation to facilitate bowel emptying, and may complain of dyspareunia or sexual dysfunction [1]. So, despite not being a life-threatening condition, POP significantly affects quality of life. Studies have shown that women have a lifetime risk of 12.6% to undergo surgical correction for POP by the age of 80 years, indicating a high burden on the healthcare system [2].

Surgical management of posterior compartment prolapse comprises a wide variety of techniques, and thus remains non-standardized, lacking a clear consensus on the optimal treatment [1, 3]. Most well-designed randomized controlled trials have focused on anterior and apical repairs; therefore, these repairs are best supported by literature; however, studies on the posterior compartment are still insufficient [4]. Traditionally, native tissue repair (colporrhaphy) is commonly practiced, either as midline fascial plication or site-specific repair [1]. The high rates of recurrence after native tissue repair encouraged the development and application of both synthetic mesh and biologic graft materials to augment and improve the durability of surgical treatment of prolapse [5]. Although transvaginal permanent mesh has a lower recurrence rate than traditional colporrhaphy does, the overall associated risks (mesh exposure and related morbidity) limit its use [3], and in April 2019, the Food and Drug Administration (FDA) ordered mesh manufacturers to stop selling devices for transvaginal repair of pelvic organ prolapse in the United States. Based on the review of available evidence, the FDA continues to believe the benefits do not outweigh the risks of surgical mesh placed transvaginally to treat POP [4, 5]. As for the biologic grafts and absorbable meshes, evidence so far does not support their use for the management of transvaginal prolapse, according to the Cochrane review [3].

In this context, amniotic membrane (AM) has emerged as a promising biological scaffold because of its anti-inflammatory, antimicrobial, angiogenic, and regenerative properties [6]. Although AM has been increasingly used in wound healing, ophthalmology, and some gynecologic procedures due to its anti-inflammatory and regenerative properties, its application in posterior compartment prolapse repair remains largely unexplored [7].

This study aimed to assess the safety, feasibility and effectiveness of the use of amniotic membrane as a graft in posterior colporrhaphy.

## Methods

This pilot, one-arm clinical trial was conducted at Ain Shams University Hospital (ASUH), Cairo, Egypt, from November 2023 to August 2025. The study protocol was approved by the ethical committee of the faculty of Medicine, Ain Shams University (FMASU MD 81/2023). The study was conducted in accordance with the Declaration of Helsinki and registered at ClinicalTrials.gov *(NCT06117670).* Informed consent was obtained from all participants before recruitment in the study. All data was collected confidentially.

We included 30–60 -year-old women who were diagnosed with posterior compartment prolapse and were scheduled for surgical correction. The exclusion criteria were pregnancy, anemia (hemoglobin less than 10 g/dL), uncontrolled medical disorders and immunocompromised patients. Examples as patients with uncontrolled diabetes mellitus, as well as uncontrolled bronchial asthma, patients on regular high dose steroids, and patients receiving cytotoxic medications were excluded. Also, we’ve excluded patients who had previous surgery for POP.

The patients were recruited from the outpatient gynecology clinic of ASUH. History-taking, physical examination, and investigations were performed to identify eligible patients as follows:

- History-taking, including age, menstrual history, medical history, and surgical history as well as symptoms score was obtained via the Pelvic Organ Prolapse Distress Inventory 6 (POPDI-6) part of the Pelvic Floor Distress Inventory (PFDI) Short Form 20. The PFDI is a validated questionnaire used to assess how lower urinary tract, lower gastrointestinal tract, and pelvic organ prolapse symptoms affect the quality of life of women who suffer from disorders of the pelvic floor. It comprises 3 scales: the Urinary Distress Inventory, the Pelvic Organ Prolapse Distress Inventory (POPDI), and the Colorectal-Anal Distress Inventory [8]. The POPDI-6 is a short, validated form of the POPDI, comprising 6 questions about the presence of prolapse symptoms and how much they bother the respondent over the last 3 months (higher scores indicate more bothersome symptoms) [8]. Additionally, sexual function was assessed via the Female Sexual Function Index Questionnaire (FSFI). The FSFI is a brief measure of female sexual arousal and other relevant domains of sexual functioning in women, namely: desire, lubrication, orgasm, satisfaction, and pain, over the past month (higher scores indicate better sexual function) [9, 10]. For both questionnaires, there’s currently no available validated Arabic version, so the physician collecting the data used the English version and translated it while interviewing the patients.
- Physical examination included general examination, abdominal examination, pelvic examination and pelvic organ prolapse quantification (POP-Q) staging.
- Routine investigations included complete blood count and other preoperative tests as needed.

### Gamma-irradiated sterilized amnion grafts

Gamma-irradiated sterilized air-dried amnion grafts were prepared and obtained from the National Center for Radiation Research and Technology, Egyptian Atomic Energy Authority. The human amniotic membranes there were obtained from placentae of healthy women who were undergoing elective cesarean section, and who tested negative for syphilis, hepatitis B virus, hepatitis C virus, and human immunodeficiency virus, after giving informed consent. Processing was then performed to obtain clean amniotic membranes free of blood clots and debris, according to methodology published previously by Marsit et al. [11] in accordance with the International Atomic Energy Agency standards. The clean amniotic membranes were air-dried in a laminar flow cabinet to remove 95% of the moisture. Processed air-dried amniotic membranes were cut into 5*5 cm^2^ pieces and then sterilized by exposure to 25 kGy of cobalt 60 gamma rays. Irradiated AM patches were validated for sterility assurance using Method VDmax 25 according to ISO in sterilization of healthcare products - radiation [11]. Ready-to-use irradiated AM patches can be stored at room temperature for up to five years.

### Surgical procedure (posterior colporrhaphy with application of the amniotic membrane graft)

After providing written informed consent, eligible patients underwent the surgical procedure as follows. All patients were given standard preoperative antibiotic prophylaxis (second generation cephalosporin) and the vagina was prepped with povidone-iodine as a part of standard surgical disinfection.

The patient was placed in the lithotomy position. A midline incision was extended from the perineal body to the cephalad border of the rectocele. Rectovaginal fascia was mobilized from the vaginal epithelium, with the dissection extended caudally from the introitus, cephalad to the yellow adipose tissue of the cul-de-sac peritoneum, and laterally to the levator and obturator fascia on the pelvic sidewall. The ready-packed sterilized AM graft was implanted between the rectovaginal fascia and the vagina, with the epithelial side up facing the posterior vaginal wall. It was sutured along the dissected rectovaginal fascia using Vicryl 3 − 0 sutures. The excess AM graft was trimmed off. Then, the rectovaginal fascia was plicated with interrupted sutures using Vicryl 0 sutures. The cephalad rectovaginal septum was reattached to the ipsilateral uterosacral ligament, while the caudal portion of the rectovaginal septum was reattached to the perineal body. When a defective perineal body was present (patients with short perineal body “pb” less than or equal to 2 cm), perineorrhaphy was performed along with the posterior colporrhaphy. The superficial muscles of the perineum were plicated in the midline, and the vaginal mucosa and skin were closed as in an episiotomy repair using Vicryl 2 − 0 sutures.

### Postoperative instructions and care

A vaginal compression pack was left and removed the following day. Patients were instructed to avoid using tampons for 6 weeks and avoid sexual intercourse for at least 6 weeks.

Patients were advised to avoid constipation by drinking plenty of water and fluids, eating fruit and vegetables, also, any constant cough is to be treated promptly. Additionally, we asked the patients to avoid heavy lifting to reduce the risk of prolapse recurrence. Postoperative clinical assessment was performed via POP-Q.

### Follow-up schedule

All patients were followed up at 3 and 6 months (POPDI-6, FSFI, and POP-Q). Data was collected and recorded.

### Outcomes

Our primary outcome was clinical improvement, anatomical assessment by the postoperative POP-Q; and subjective improvement assessed by POPDI-6 score at 3 and 6 months postoperatively. *Anatomical success* implies improvement in the POP-Q stage (at least one stage improvement from baseline). *Anatomical failure* is either worsening or no change in the POP-Q stage compared to baseline. *Recurrence* implies worsening of the POP-Q stage and/or worsening of the POPDI-6 or FSFI scores (*symptomatic recurrence*) after a period (one month minimum) of initial improvement. *Retreatment/reoperation* is defined as any prolapse surgery done within 6 months, related to the compartment affected at baseline assessment. The secondary outcomes included the occurrence of infection or wound dehiscence within 6 months; occurrence of intraoperative complications such as bladder or bowel injury; and the sexual function as assessed by FSFI questionnaire at 3 and 6 months postoperatively. *Vaginal infection/vaginitis* was primarily clinically diagnosed by symptoms of vaginal itching, discomfort, soreness, abnormal vaginal discharge; and signs as redness, thick curd-like or grey frothy foul-smelling vaginal discharge. According to the local protocol, treatment was initiated based on this clinical diagnosis by local antifungal suppositories once daily for one week. If there was no response, then a vaginal swab was taken for culture and sensitivity, and the treatment was prescribed according to the culture results. *Graft-related infection* was diagnosed based on general signs of sepsis including fever, rigors, and anemia, coupled with local manifestations such as exposed graft material, discharging sinus, swelling, pain, erythema, warmth, or purulent discharge overlying the graft.

### Sample size justification

This was a pilot one-arm clinical trial, exploring a novel application of the amniotic membrane in clinical practice and testing its feasibility and safety. We enrolled a sample of 34 patients through convenience sampling, to reach a final of 25 patients present for analysis.

### Statistical analysis

The collected data was revised, coded, tabulated, and introduced to a PC using Statistical Package for Social Science (SPSS^®^ 27). Descriptive statistics were calculated for all variables, with continuous variables presented as means and standard deviations, and categorical variables presented as frequencies and percentages. For the analysis of continuous outcome measures (POPDI-6 scores and FSFI scores) across multiple time points (baseline, 3 months, and 6 months), repeated measures analysis of variance (ANOVA) was employed to assess changes over time. For repeated-measures analyses, the assumption of sphericity was assessed using Mauchly’s test. The assumption of sphericity was violated, the Greenhouse–Geisser correction was applied to the degrees of freedom. For the analysis of ordinal categorical variables (POP-Q stage) across the three time points, the Friedman test was used. Complications and adverse events were analyzed using descriptive statistics, with frequencies and percentages reported for categorical outcomes. Statistical significance was set at *p* < 0.05 for all analyses.

## Results

**Fig. 1:**
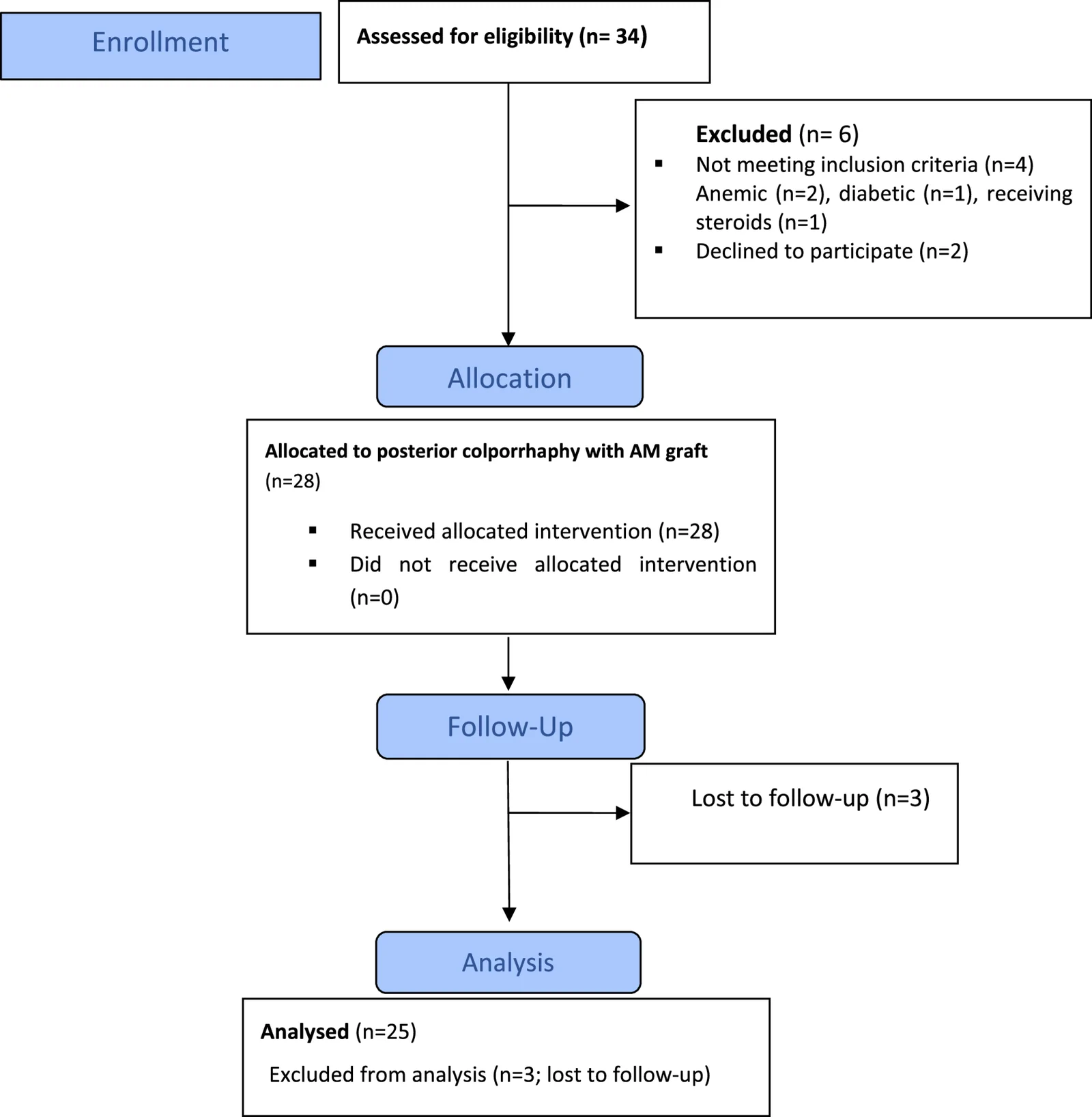
Participant flow diagram showing the recruitment and follow-up of patients during the study

Thirty-four women diagnosed with posterior compartment prolapse attending at ASUH for surgical correction were recruited from the outpatient gynecology clinic of ASUH and assessed for eligibility; of which, 28 women were enrolled and allocated to undergo posterior colporrhaphy with AM graft. Follow-up was conducted at 3months and 6months postoperative; but 3 patients didn’t show up and were lost for follow-up. The process of recruitment and follow-up of the patients during the study is shown in the participant flow diagram (Fig. 1).

### Section I: Baseline characteristics, procedures done and feasibility

The analysis included 25 participants with a mean age of 45.08 ± 6.41 years. The majority were premenopausal (92%, *n* = 23), with parity ranging from 2 to 6 deliveries, most commonly 3 deliveries (32%, *n* = 8). The majority (68%, *n* = 17) had stage II posterior compartment prolapse according to POP-Q, as shown in Table 1. Although four patients (16%) had stage I prolapse, they opted for surgical correction because they suffered bothersome symptoms. Five patients (20%) had associated anterior compartment prolapse stage II and underwent concomitant anterior colporrhaphy; while two patients (8%) had associated apical compartment prolapse and underwent concomitant abdominal sling operation. Regarding perineorrhaphy, 21 patients (84%) had short perineal body (pb less than or equal to 2 cm) and had concomitant perineorrhaphy done. As for other concomitant surgeries not related to prolapse, four patients underwent concomitant vaginal hysterectomy, and one patient had laparoscopic ovarian cystectomy. The full list of POP-Q examination points (baseline, 3 months postoperative, and 6 months postoperative) as well as the concomitant surgeries can be found in the supplementary material (additional file 1).

**Table 1:** Demographic and baseline clinical characteristics of the participants

		Mean	SD
Age		45.08	6.41
		N	%
Menopause	No	23	92%
	Yes	2	8%
Parity	2	4	16%
	3	8	32%
	4	4	16%
	5	5	20%
	6	4	16%
Baseline POP-Q stage	0	0	0%
	I	4	16%
	II	17	68%
	III	2	8%
	IV	2	8%

	Mean	SD	95% CI of the mean
Baseline POPDI-6 score	68.00	14.52	62.01–73.99
Baseline FSFI score	11.75	3.55	10.29–13.22

Recruitment of patients was a bit difficult; because it’s uncommon to find “isolated” posterior compartment prolapse. Most of the cases had combined defects, so we had to include some cases with other compartment defects, and did concomitant corrective surgeries as described earlier.

The AM graft was successfully implanted in all cases. It was technically feasible, but it requires gentle handling with non-toothed forceps and using small-curved needles with fine 3 − 0 sutures to avoid tissue disruption.

### Section II: Clinical outcomes and follow-up at 3 and 6 months

**Fig. 2:**
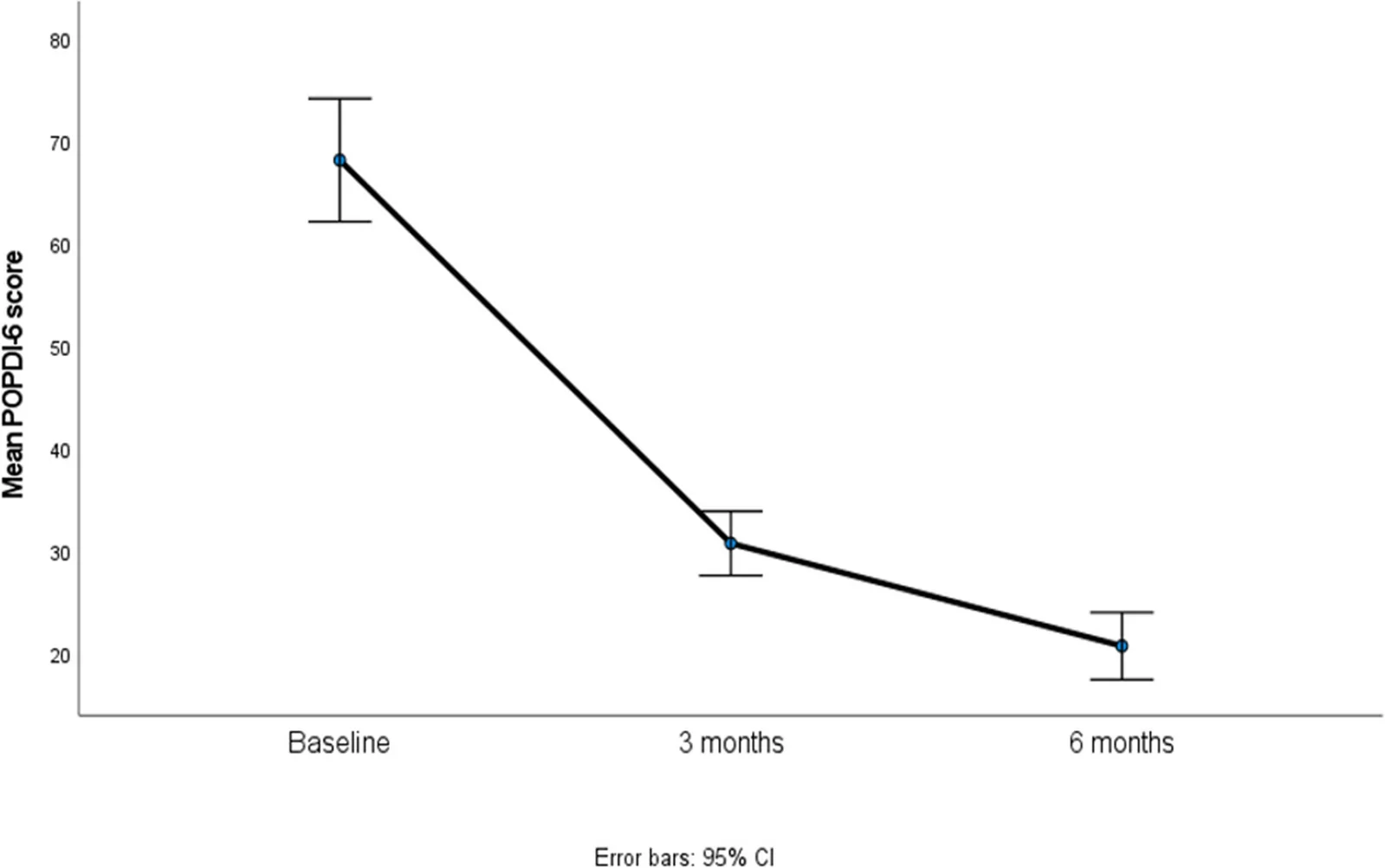
Mean POPDI-6 score changes over time

The POPDI-6 score improved from baseline (68 ± 14.52) to 3 months (30.66 ± 7.60) and 6 months (20.67 ± 7.93) after surgery, as shown in Table 2; Fig. 2. Compared to baseline, the mean reduction in symptom score was 53.65% ± 12.04% in 3 months (95% CI: 48.68–58.62%), and 68.22% ± 14.54% in 6 months (95% CI: 62.22–74.23%).

**Table 2:** POPDI-6 score changes over time

	Mean	SD	95% CI of the mean	Repeated measure ANOVA		
				F	p value	sig.
Baseline POPDI-6 score	68.00	14.52	62.01–73.99	198.97	< 0.001	S
POPDI-6 score at 3 months	30.66	7.60	27.53–33.8			
POPDI-6 score at 6 months	20.67	7.93	17.39–23.94			

All pairwise comparisons were significant with *p* < 0.001

**Fig. 3:**
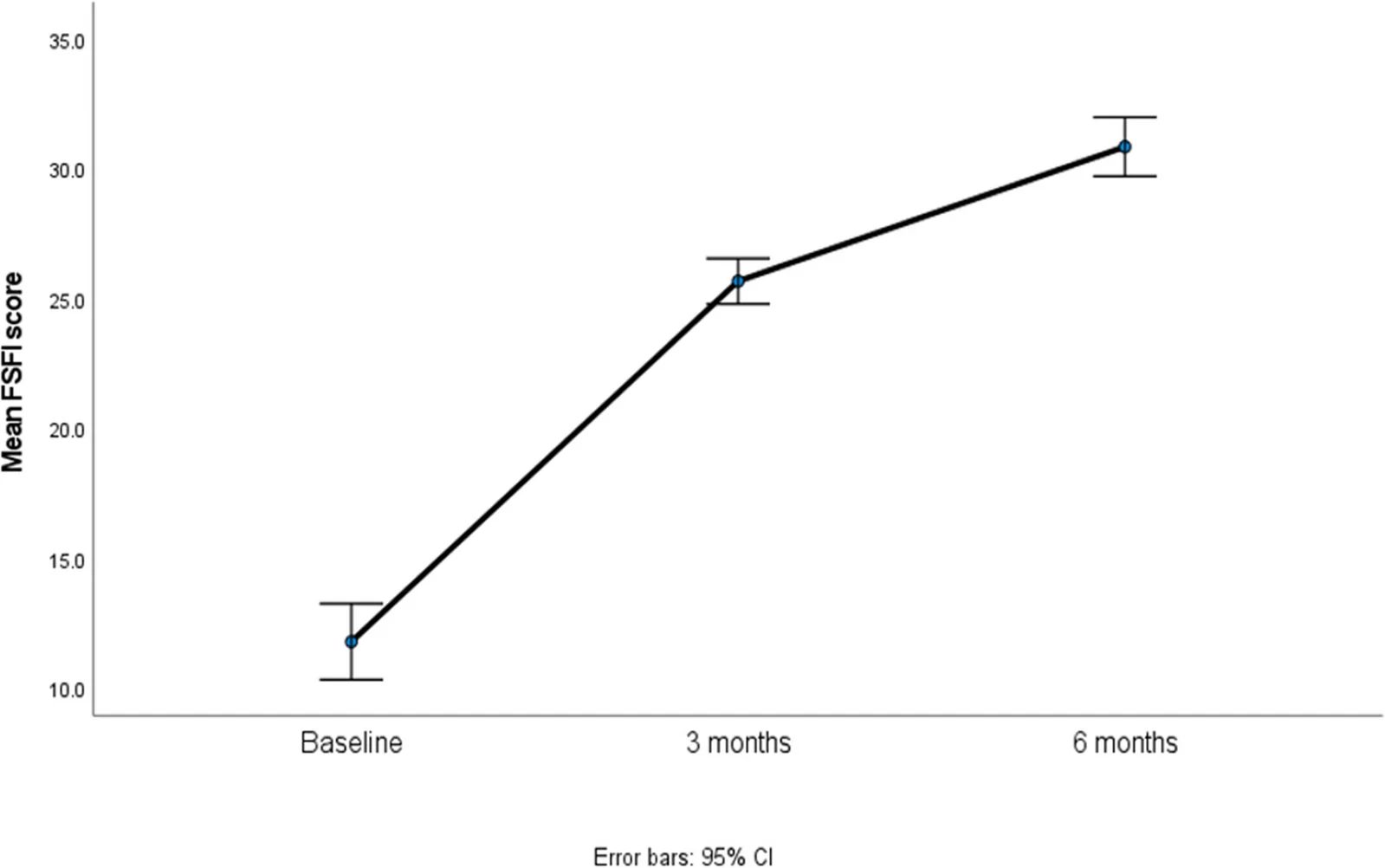
Mean FSFI score changes over time

Regarding the sexual function (FSFI) score, it increased from baseline (11.75 ± 3.55) to 3 months (25.63 ± 2.12) and 6 months (30.80 ± 2.75) after surgery, as shown in Table 3; Fig. 3.

**Table 3:** FSFI score changes over time

	Mean	SD	95% CI of the mean	Repeated measure ANOVA		
				F	p value	sig.
Baseline FSFI score	11.75	3.55	10.29–13.22	363.56	< 0.001	S
FSFI score at 3 months	25.63	2.12	24.75–26.50			
FSFI score at 6 months	30.80	2.75	29.66–31.94			

All pairwise comparisons were significant with *p* < 0.001

POP-Q staging showed some improvement, with 68% of patients initially at Stage II and 16% at Stages III-IV, whereas at 3 and 6 months, 80% achieved Stage 0 (no prolapse), with 20% at Stage I, as shown in Table 4. The change in the specific points (Aa, Ba, Ap, Bp, C, D, pb, gh, tvl) over time is presented in Table 5; showing improvements both in the anterior and posterior compartment points, as well as significant changes in genital hiatus (gh) and perineal body (pb).

**Table 4:** POP-Q staging preoperatively and upon follow-up

POP-Q	Baseline		3 months		6 months		Friedman test		
	*N*	%	N	%	N	%	test value	p value	sig.
Stage 0	0	0.0%	20	80.0%	20	80.0%	50	< 0.001	S
Stage I	4	16.0%	5	20.0%	5	20.0%			
Stage II	17	68.0%	0	0.0%	0	0.0%			
Stage III	2	8.0%	0	0.0%	0	0.0%			
Stage IV	2	8.0%	0	0.0%	0	0.0%			

**Table 5:** POP-Q point measurements across time points

Variable		Mean	SD	95% CI of the mean	Repeated measure ANOVA		
					**F**	**p value**	**sig.**
Aa	Baseline	-2.04^a^	1.43	-2.63 to -1.45	7.227	0.002	S
	3 months	-2.76^b^	0.44	-2.94 to -2.58			
	6 months	-2.76^b^	0.44	-2.94 to -2.58			
Ba	Baseline	-1.72^a^	1.70	-2.42 to -1.02	7.271	0.002	S
	3 months	-2.64^b^	0.49	-2.84 to -2.44			
	6 months	-2.64^b^	0.49	-2.84 to -2.44			
Ap	Baseline	0.08^a^	1.35	-0.48 to 0.64	160.673	< 0.001	S
	3 months	-2.84^b^	0.37	-2.99 to -2.69			
	6 months	-2.84^b^	0.37	-2.99 to -2.69			
Bp	Baseline	0.96^a^	1.86	0.19 to 1.73	140.069	< 0.001	S
	3 months	-2.80^b^	0.41	-2.97 to -2.63			
	6 months	-2.80^b^	0.41	-2.97 to -2.63			
C	Baseline	-5.72^a^	2.62	-6.80 to -4.64	2.000	0.146	NS
	3 months	-6.32^a^	1.07	-6.76 to -5.88			
	6 months	-6.32^a^	1.07	-6.76 to -5.88			
D	Baseline	-7.10^a^	2.77	-8.36 to -5.83	1.000	0.377	NS
	3 months	-7.57^a^	1.16	-8.10 to -7.04			
	6 months	-7.57^a^	1.16	-8.10 to -7.04			
gh	Baseline	3.38^a^	0.67	3.11 to 3.65	13.947	< 0.001	S
	3 months	2.92^b^	0.40	2.75 to 3.09			
	6 months	2.96^b^	0.43	2.78 to 3.14			
pb	Baseline	1.74^b^	0.71	1.45 to 2.03	94.074	< 0.001	S
	3 months	3.48^a^	0.49	3.28 to 3.68			
	6 months	3.48^a^	0.47	3.29 to 3.67			
TVL	Baseline	8.24^a^	1.23	7.73 to 8.75	2.519	0.091	NS
	3 months	8.12^a^	1.24	7.61 to 8.63			
	6 months	8.04^a^	1.21	7.54 to 8.54			

F: repeated measures ANOVA test statistic, sharing the same letter means they are not significantly different

### Section III: Safety and adverse events

No intraoperative complications or cases of wound dehiscence occurred. Vaginal infection was common, affecting 76% of the patients (*n* = 19). However, it was mild vaginitis, requiring local treatment only. Diagnosis of vaginitis was based on clinical symptoms and signs (vaginal itching, soreness, redness, thick curd-like foul-smelling vaginal discharge). We haven’t noticed any recognizable time-pattern for presentation, but only variable presentation within the first month postoperative. No vaginal swabs/cultures were obtained, as the patients responded to local treatment per hospital protocol (antifungal “miconazole” vaginal suppository once daily for one week). There were no cases of graft-related infection.

## Discussion

This pilot single-arm clinical trial was conducted on women with symptomatic posterior compartment prolapse (POP-Q stages I–IV) to assess the feasibility, effectiveness and safety of posterior colporrhaphy using gamma-irradiated amniotic membrane as a graft. Our study revealed a reduction in POPDI-6 scores from 68.00 ± 14.52 at baseline to 20.67 ± 7.93 at 6 months (*p* < 0.001), with a mean percentage reduction of 68.22% ± 14.54%. Sexual function score improved from 11.75 ± 3.55 to 30.80 ± 2.75 at 6 months postoperatively. Anatomical assessment revealed that 80% of patients achieved POP-Q stage 0, whereas 20% reached stage I by 6 months. No serious complications (wound dehiscence, visceral injury) were reported; however, 76% of patients experienced mild vaginitis.

The use of the amniotic membrane as an adjunctive therapy in pelvic organ prolapse surgery was first described by Seifeldin in 2015 [12]. He used fresh amniotic membrane as a graft in 21 patients undergoing anterior and/or posterior vaginal repair. On follow-up (a minimum of 30 days), he reported that there were no infections or rejections; no bleeding or wound dehiscence; and 3 patients had de novo urinary urgency which was medically treated. However, there’s no data regarding POP-Q staging or patient-oriented outcomes such as symptom score or sexual satisfaction [12].

In comparison, Elkassas et al. used gamma-irradiated sterilized amniotic membrane as a graft in patients undergoing classical repair. They performed a prospective clinical trial involving two groups of patients with anterior and posterior vaginal wall prolapse. One group (20 patients) underwent vaginal repair using gamma-irradiated amniotic membrane as a graft, whereas the other group (20 patients) underwent traditional anterior and posterior colporrhaphy without using amniotic membrane [13]. They followed the patients for 6 months and reported upon postoperative infection, wound healing, fibrosis formation, dyspareunia, and recurrence. There was no significant difference between the groups in any of the outcomes except for dyspareunia, which was significantly less frequent in the AM group [13]. Despite reporting patient-relevant outcomes, they did not use objective assessments or standardized questionnaires and scores.

Recently, a growing body of evidence has described the regenerative properties of the amniotic membrane, with potential applications in different fields including gynecology [6, 7]. Amniotic membrane can be used as a graft during McIndoe vaginoplasty in patients with Mullerian agenesis resulting in successful recovery and satisfactory sexual performance. This was evidenced both by biopsy, which revealed proper vaginal epithelization without scars or adhesions, and by improvement of the FSFI score postoperatively [7, 14].

Vaginal mesh erosion is another entity where AM can be of great benefit. Complex vaginal mesh erosions, recurrent ulcerations, or mesh exposure are sometimes difficult to heal, or heal with scarring and/or vaginal agglutination. The layers of AM, comprising pluripotent cells, nutrients, growth factors, extracellular matrix proteins, and cytokines, serve as an excellent microenvironment that promotes cell growth and differentiation without scarring or an excess inflammatory response. This is ideal for promoting the healing of ulcers and complex erosions [7]. This phenomenon was explored in the clinical trial by Lau et al., as they used amniotic membrane as a graft after surgical mesh excision in 8 patients suffering from complex vaginal mesh erosions in whom conservative therapy failed. Seven patients recovered well without recurrence. Only one patient experienced recurrent erosion, which was smaller in size, and was subsequently repaired successfully [15].

Another promising role for AM is as an adjunctive therapy in patients with Asherman syndrome. Amniotic membrane can be applied as a graft on the endometrial lining after surgical resection of the intrauterine adhesions. The AM promotes endometrial proliferation, thereby preventing the recurrence of adhesions, and restoring menstrual flow, and in some cases, successful pregnancy has been reported [7, 16].

Different clinical trials have utilized the amniotic membrane in different forms. Some have used fresh AM, others have used cryopreserved AM, while others have used lyophilized gamma-irradiated AM [6, 7]. The advantage of air-dried AM is being an easy and inexpensive technique for storage and reuse. Although air drying and gamma-irradiation may affect the cellular component of the AM, studies have shown that both fresh and sterilized air-dried AMs yield good clinical outcomes and positive healing results [6]. In our study, we used air-dried gamma-irradiated AM to ensure sterility of the product and ease of use.

During our follow-up through 3 and 6 months postoperative, no cases of wound dehiscence, erosion or tissue rejection were observed. These early findings support the presumed safety of using the amniotic membrane as a graft. Similarly, Seifeldin reported that there were no rejections or cases of wound dehiscence among patients during follow-up (four patients were followed up to 180 days) [12]. Also, the study by Elkassas et al. did not observe any cases of wound dehiscence or rejection during the 6 months follow-up [13]. To date, no long-term outcomes have been reported regarding the use of amniotic membrane graft during colporrhaphy. However, Lau et al. reported long-term results of using amniotic membrane as a graft during surgical repair of complex vaginal mesh erosions. After an average of 27 months (range: 6–45 months) of follow-up, seven out of eight patients were successfully treated. No immunosuppressants were given, and no immune graft rejection was encountered [15].

Although we observed a high vaginal infection rate (76%) in our study, it was mild vaginitis (in the form of vaginal discharge and mild itching/discomfort), and all the patients responded to standard local treatment. This warrants further investigation into the cause and whether AM application affects the normal vaginal flora. The AM is known for its inherent antimicrobial, anti-inflammatory, and immunomodulatory properties [6, 7]. Prior studies showed that the AM antimicrobial effect involves different molecules; some of them are α and β defensins, which possess anti-bacterial, anti-viral, and antifungal activity, particularly defensin β3, which possess a significant anti-bacterial effect [7]. This antimicrobial effect is thought to be maintained even after gamma-irradiation [6]. Despite being advantageous for wound healing, this effect may alter the local vaginal environment, causing imbalance in the normal vaginal flora, which may manifest as vaginitis. Our findings contradict the results of Elkassas et al., who reported no postoperative infections in patients managed with amniotic graft in posterior colporrhaphy [13]. Seifeldin reported only one case of delayed wound healing (out of 12 cases in which an amniotic graft was used in posterior colporrhaphy), but no other infection or wound dehiscence occurred [12]. As for Lau et al., 4 out of 8 (50%) patients who underwent surgical management of complex vaginal mesh erosions with amniotic graft complained of temporary vaginal discharge after surgery, but none of the patients experienced wound infection [15]. In our opinion, these discrepancies in the reported rates of infection are due to a lack of standardized definitions/diagnoses of infection. This may include the following: altered/excessive vaginal discharge with symptoms such as itching/discomfort; generalized sepsis with fever and a systemic inflammatory response; or delayed wound healing or wound dehiscence; these are all possible outcomes, unfortunately not always explicitly reported. Another possible explanation for this discrepancy in reported rates of vaginal infection is the difference in the method of preparation of the amniotic grafts. Seifeldin used fresh amniotic membranes [12]; while Lau et al. used cryopreserved amniotic membrane grafts [15]. Elkassas et al. reported using gamma irradiated, freeze-dried amniotic membrane grafts, but the detail of preparation is not well-explained [13]. Nevertheless, the effect of amniotic membrane on the vaginal microbiota should be further investigated in future studies, taking into account the exact method of preparation including the irradiation dose and cryopreservation/thawing process.

### Strengths and limitations

Our study presents a standardized evaluation of a novel technique, using gamma-irradiated air-dried AM as a graft in posterior colporrhaphy. We used objective assessment (POP-Q classification) for proper staging of the degree of prolapse. Additionally, we reported patient-relevant outcomes via the standardized symptom score index (POPDI-6) and measured sexual function via the FSFI score. Moreover, we assessed the immediate and short-term safety results of the technique, monitoring the patients for any sign of wound complication/rejection, at 3 months and 6 months, although long-term effects and safety results are not yet available. The use of the sterilized, shelf-stable form of AM increases the real-world applicability of this technique.

Despite these favorable outcomes, our study has several limitations. First, it is still a pilot study, with no comparison group. We cannot guarantee whether the improvement observed in the patients was due to the colporrhaphy itself or due to the added effect of support and regenerative power of the AM. A randomized clinical trial with head-to-head comparisons between traditional colporrhaphy and AM-grafted colporrhaphy is needed. This should explore the differences in efficacy, side effects, and whether the observed vaginal infection rate (76%) is attributed to AM application. Additionally, we need to follow patients for a longer period of time to detect the recurrence rate and compare it in both groups in the long run. Another major limitation in our study is that no formal sample size calculation or power analysis was conducted; as we were aiming to test feasibility and initial safety as a pilot study. Future studies with head-to-head comparisons will employ a proper sample size based on formal calculation and power analysis.

Our study paves the way for future directions. In addition to performing head-to-head comparative studies, we need to test and compare the cost-effectiveness of AM grafts (fresh, cryopreserved, and air-dried). Moreover, future trials may evaluate the regenerative potential of this technique in recurrent cases.

## Conclusions

Our study demonstrated that posterior colporrhaphy using gamma-irradiated amniotic membrane grafts is technically feasible, with no serious adverse events. The procedure was associated with improvements in prolapse-related symptoms, sexual function, and clinical examination, with no recurrences or graft-related complications during the follow-up period. Future randomized controlled clinical trials are necessary to compare this technique with traditional colporrhaphy.

## Supplementary Information

Additional file 1: POP-Q dataset. This is the data of the POP-Q examination of the participants at baseline, 3months, and 6months; along with the concomitant surgical procedures done.

Additional file 2: Dataset. This is the data of the participants that’s used and analyzed in our research.

## Data Availability

The dataset used and analyzed during the current study is available as a supplementary file (Additional file1; Additional file2).

## Abbreviations

AM: Amniotic membrane
ASUH: Ain Shams University Hospital
FSFI: Female Sexual Function Index
PFDI: Pelvic Floor Distress Inventory
POPDI-6: Pelvic Organ Prolapse Distress Inventory 6
POP-Q: Pelvic organ prolapse quantification
