# Supplementary material for: Gamma-irradiated amniotic membrane graft as an adjuvant to posterior colporrhaphy: an exploratory prospective single-arm clinical trial
Source: BMC Womens Health. 2026 Oct 17;26:532. doi: 10.1186/s12905-026-04919-9 (PMC13675953; doi:10.1186/s12905-026-04919-9)
Supplement: Supplementary file 1 [file 12905_2026_4919_MOESM2_ESM.docx]

| **ID** | **Age** | **Menopause** | **Parity** | **Baseline POPDI-6 score** | **Baseline POP-Q Stage** | **Baseline FSFI score** | **Intraoperative complications** | **3 months POPDI-6 score** | **3 months POP-Q Stage** | **3 months FSFI score** | **6 months POPDI-6 score** | **6 months POP-Q stage** | **6 months FSFI score** | **Wound dehiscence** | **Vaginal infection** |
| --- | --- | --- | --- | --- | --- | --- | --- | --- | --- | --- | --- | --- | --- | --- | --- |
| 1 | 50 | 0 | 5 | 33.33 | 1 | 16.6 | 0 | 25.00 | 0 | 28.4 | 25.00 | 0 | 28.4 | 0 | 1 |
| 2 | 45 | 0 | 4 | 54.17 | 1 | 11.6 | 0 | 33.33 | 0 | 25.2 | 16.67 | 0 | 27.2 | 0 | 1 |
| 3 | 44 | 0 | 3 | 62.50 | 2 | 17.3 | 0 | 33.33 | 1 | 23.7 | 8.33 | 1 | 25.2 | 0 | 1 |
| 4 | 47 | 0 | 2 | 79.17 | 2 | 14.1 | 0 | 45.83 | 0 | 22.2 | 33.33 | 0 | 34.3 | 0 | 0 |
| 5 | 39 | 0 | 3 | 41.67 | 2 | 12.2 | 0 | 20.83 | 0 | 23.4 | 20.83 | 0 | 34.3 | 0 | 0 |
| 6 | 42 | 0 | 2 | 66.67 | 2 | 11.9 | 0 | 45.83 | 0 | 24.8 | 33.33 | 0 | 33.1 | 0 | 1 |
| 7 | 35 | 0 | 5 | 58.33 | 2 | 10.7 | 0 | 33.33 | 0 | 27 | 25.00 | 0 | 30.6 | 0 | 1 |
| 8 | 33 | 0 | 4 | 58.33 | 2 | 11 | 0 | 20.83 | 0 | 26.9 | 8.33 | 0 | 35.4 | 0 | 0 |
| 9 | 45 | 0 | 6 | 83.33 | 4 | 2 | 0 | 33.33 | 1 | 23.2 | 25.00 | 1 | 29.2 | 0 | 1 |
| 10 | 58 | 1 | 3 | 91.67 | 3 | 2 | 0 | 41.67 | 1 | 21 | 29.17 | 1 | 32.3 | 0 | 0 |
| 11 | 48 | 0 | 2 | 62.50 | 2 | 10.3 | 0 | 33.33 | 0 | 24.5 | 12.50 | 0 | 26.7 | 0 | 1 |
| 12 | 34 | 0 | 3 | 50.00 | 1 | 14.2 | 0 | 20.83 | 0 | 28.4 | 8.33 | 0 | 32.5 | 0 | 1 |
| 13 | 50 | 0 | 3 | 87.50 | 4 | 9 | 0 | 37.50 | 1 | 23.3 | 16.67 | 1 | 27.4 | 0 | 0 |
| 14 | 55 | 1 | 6 | 79.17 | 3 | 10 | 0 | 33.33 | 1 | 25.8 | 20.83 | 1 | 28 | 0 | 1 |
| 15 | 49 | 0 | 5 | 66.67 | 1 | 12.4 | 0 | 33.33 | 0 | 28.8 | 25.00 | 0 | 31.8 | 0 | 1 |
| 16 | 51 | 0 | 3 | 62.50 | 2 | 11.9 | 0 | 20.83 | 0 | 27.3 | 16.67 | 0 | 30.7 | 0 | 1 |
| 17 | 47 | 0 | 6 | 70.83 | 2 | 16.7 | 0 | 25.00 | 0 | 27.4 | 25.00 | 0 | 30.7 | 0 | 0 |
| 18 | 41 | 0 | 4 | 66.67 | 2 | 11.8 | 0 | 20.83 | 0 | 27.2 | 12.50 | 0 | 29.4 | 0 | 1 |
| 19 | 36 | 0 | 5 | 91.67 | 2 | 13.1 | 0 | 25.00 | 0 | 24.1 | 4.17 | 0 | 30.3 | 0 | 1 |
| 20 | 45 | 0 | 3 | 66.67 | 2 | 12.7 | 0 | 29.17 | 0 | 27.6 | 25.00 | 0 | 33.1 | 0 | 1 |
| 21 | 47 | 0 | 2 | 83.33 | 2 | 11.7 | 0 | 25.00 | 0 | 25.8 | 25.00 | 0 | 31.8 | 0 | 1 |
| 22 | 51 | 0 | 3 | 75.00 | 2 | 12.4 | 0 | 37.50 | 0 | 23.8 | 25.00 | 0 | 28.3 | 0 | 1 |
| 23 | 40 | 0 | 6 | 75.00 | 2 | 12.3 | 0 | 33.33 | 0 | 26.4 | 25.00 | 0 | 31.5 | 0 | 1 |
| 24 | 50 | 0 | 5 | 70.83 | 2 | 12.8 | 0 | 25.00 | 0 | 27.1 | 25.00 | 0 | 33.1 | 0 | 1 |
| 25 | 45 | 0 | 4 | 62.50 | 2 | 13.1 | 0 | 33.33 | 0 | 27.4 | 25.00 | 0 | 34.7 | 0 | 1 |

**Title of the manuscript:** Gamma-irradiated Amniotic Membrane Graft as an adjuvant to Posterior Colporrhaphy. An exploratory prospective single-arm clinical trial.

**Journal name:** BMC Women's Health

**Corresponding author:** Rania Hassan Mostafa Ahmed; M.D. **ORCID:** 0000-0002-9918-2363 **e-mail:** raneyah@med.asu.edu.eg
